# The first of a series of articles dedicated to the 60^th^ anniversary of the Brazilian Society of Genetics (SBG)

**DOI:** 10.1590/1678-4685-GMB-2016-00030002

**Published:** 2016

**Authors:** Francisco Mauro Salzano, Fabrício Rodrigues dos Santos, Klaus Hartfelder, Carlos F.M. Menck

**Affiliations:** 1 Departamento de Genética, Instituto de Biociências, Universidade Federal do Rio Grande do Sul – UFRGS, Porto Alegre, RS, Brazil.; 2 Departamento de Biologia Geral, Instituto de Ciências Biológicas, Universidade Federal de Minas Gerais – UFMG, Belo Horizonte, MG, Brazil.; 3 Departamento de Biologia Celular e Molecular e Bioagentes Patogênicos, Faculdade de Medicina de Ribeirão Preto, Universidade de São Paulo – USP, Ribeirão Preto, SP, Brazil.; 4 Departamento de Microbiologia, Instituto de Ciências Biomédicas, Universidade de São Paulo – USP, São Paulo, SP, Brazil.

In July of 1955, a group of around 20 Brazilian geneticists got together in a meeting and took the decision to launch the Brazilian Society of Genetics (Sociedade Brasileira de Genética, SBG). This Society flourished rapidly, and today it has close to one thousand members. Since its foundation, this association of scientists has worked hard and continuously to advance the quality of research in Genetics to international levels. One among the several decisions taken 39 years ago, was that SBG should have its own society journal, to give more visibility to Genetics research done in Brazil. The initial title of the journal was Revista Brasileira de Genética, which, in 1995, was changed to Brazilian Journal of Genetics. Shortly thereafter, in 1998, its name then became Genetics and Molecular Biology, and all articles published since were exclusively in English. The decision was taken in view of the ongoing process of internationalization in the scientific publications field, and now offered a platform also for international researchers to submit and publish their results in this Open Access journal. Since its beginning, the journal succeeded to publish four issues every year, and it has and is clearly achieving its goal to be an attractive platform for good quality scientific contributions from all over the world in the broad field of Genetics, Molecular Biology and Evolution.

One year ago, as the Society was celebrating its 60^th^ anniversary, the SBG's Board of Directors and the GMB Editors decided that it would be an interesting moment to gather scientific contributions honoring the Society. A call was launched, inviting several leading Geneticists from Brazil and abroad, and the response was very positive to this initiative. Their respect to the SBG and to this journal is now manifest by high quality reviews and original articles, which will be published in a series of GMB issues, starting with the current one.

Thus, for the celebration of the 60^th^ anniversary of SBG, this issue of GMB (39.3) brings six articles, including four reviews and two original researches. Dr. Salzano offers us an in-depth review of many aspects of human evolution, including important contributions from Latin American studies. Dr. Vieira and her group review the relevance of microsatellites in plant sciences studies, discussing what they are, how they can be detected, and applied to the field. The group of Dr. Diaz provides an interesting review that covers recent findings about plant senescence mediated by abiotic and biotic stresses. Dr. Zatz and her group review their most important scientific contributions in the last thirty years on neuromuscular disorders, in terms of gene discovery, diagnosis, counseling and also therapy. The group of Dr. Pena reports on a genotype-phenotype study identifying mutations that result in the haploinsufficiency of the *NARS* gene, which may be responsible for clinical features of the Noonan Syndrome. Finally, the group of Dr. Tanuri describes a rapid test to evaluate the sensitivity of hepatitis C virus from infected patients to protease inhibitors, with potential application in clinical diagnosis.

We believe that this first and also the upcoming series of scientific articles dedicated to the 60^th^ anniversary offer a good overview on the breadth of Genetics research done in Brazil and in collaboration with international group leaders, and, together with the other published works in this and the upcoming issues, will stimulate readers and geneticists to appreciate even more this scientific society and its journal. Enjoy!

Fabrício Rodrigues dos Santos and Francisco Mauro Salzano Guest Editors of the
SBG 60 years Special Series of Articles Carlos F.M. Menck and Klaus
Hartfelder Editors of Genetics and Molecular Biology

